# Influence of Gradient Milling on Cooking and Sensory Attributes of Chinese Black Rice: Insights into Volatile Flavor Compounds

**DOI:** 10.3390/foods13213453

**Published:** 2024-10-29

**Authors:** Shuxin Ye, Qing Gao, Danxia Shi, Abel Wend-Soo Zongo, Jinsong He, Bin Li

**Affiliations:** 1 College of Food Science and Technology, Yunnan Agricultural University, Kunming 650201, China; m13628691513@163.com (S.Y.); 2015053@ynau.edu.cn (Q.G.); sdanqiu@outlook.com (D.S.); 2 College of Food Science and Technology, Huazhong Agricultural University, Wuhan 430070, China; patarbtaale@gmail.com; 3 Hubei Collaborative Innovation Centre for Industrial Fermentation, Hubei University of Technology, Wuhan 430068, China; 4 Key Laboratory of Environment Correlative Dietology, Huazhong Agricultural University, Ministry of Education, Wuhan 430070, China

**Keywords:** Yangxian black rice, milling degree, cooking properties, eating quality, volatiles

## Abstract

This study investigated the impact of gradient milling on the cooking properties and sensory characteristics of Yangxian black rice. The results showed that as the degree of milling increased, the gelatinization time decreased (36.85–23.54 min) and the water uptake ratio of whole black rice (188.29%) was significantly lower compared to that of refined grains (194.05%). Low-field nuclear magnetic resonance (NMR) was further used to monitor the water concentration and distribution of black rice during soaking and cooking. It was found that the bran layers of black rice, as a physical barrier, impeded the water penetration into the kernels for a given soaking and cooking duration. The sensory evaluation conducted by a panel of trained volunteers demonstrated a high score for all sensory attributes in slightly milled black rice, corroborating findings from the taste analyzer. Through correlation analysis of volatile components determined by gas chromatography-mass spectrometry (GC-MS), smell scores in sensory evaluation, and electronic nose response values, 2-pentyl-furan (54.84–12.72 ng/g) and guaiacol (19.39–5.51 ng/g) were found to be the predominant volatile flavor contributors in cooked black rice. Overall, this study provides valuable insight into the intricate relationship between milling degrees and the cooking properties, sensory characteristics, and volatile flavor compounds of Yangxian black rice.

## 1. Introduction

Rice (*Oryza sativa* L.), a staple food for nearly half of the global population, is one of the most important cereal crops worldwide. The harvested paddy consists of the husk, bran, embryo, and endosperm [1,2,3]. In the rice industry, brown rice is produced by removing the hull from the rough paddy while white rice undergoes additional milling and polishing (whitening process) to remove the bran [4,5,6,7]. Compared with brown rice, refined rice received higher acceptance in certain cases due to its more delicate taste and texture [8]. The milling process destroys the bran structure of grain, leading to the improvement of the whiteness levels and cooking quality of the rice while the nutrients are lost [5,7]. The concept of “Energy Saving and Loss Reduction via Moderate Processing” was introduced by China’s Grain Industry in 2011 to tackle the problem of excessive rice processing [7,9]. Therefore, investigating the changes in both sensory quality and nutritional benefits of rice during its transition from whole to refined grains under gradient milling treatments provides valuable insights for optimizing moderate rice processing to balance between taste and nutrition [4,5].

Black rice has a purple-black or black kernel color due to the presence of anthocyanin in its bran layer [6]. Under the global consensus that a healthy human diet must be rich in whole grain rather than refined grain, pigmented rice, especially black rice, has emerged as a preferred alternative staple food [10,11,12]. China is the largest producer of black rice, contributing 62% of global production [6,12]. Among its ancient varieties, Yangxian black rice has been cultivated for more than 2000 years and was historically reserved for emperors. This precious variety was designated as a Chinese national geographical indication protected product in 2006 (standard DB 61/T 1011-2018 [13]).

The increasing consumption of rice is accompanied by stricter consumer demands for rice products with premium qualities. Rice industries tend to measure the economic value of rice in terms of cooking and eating qualities that can be tested by gelatinization time (cooking duration), water uptake ratio, solids in the cooking water, and sensory characteristics [14]. The starch gelatinization induced by hydration and heating is strongly associated with the texture and eating quality of cooked rice [15]. The sensory characteristics including appearance, aroma, taste, and texture play a crucial role in consumer acceptance since rice is generally eaten without seasoning. Besides the complicated sensory test by trained panelists, the instrumental measurements of the textural attributes to predict the overall eating quality of cooked rice have met with partial success [16,17]. Although a rice taste analyzer developed in Japan correlates key constituents with “taste” scores, it failed to capture volatile flavor characteristics, especially for pigmented rice. Therefore, determining the dominant volatile flavor compound in cooked pigmented rice remains challenging.

The present work was undertaken to investigate the cooking properties and sensory characteristics of cooked black rice under gradient milling. Changes in moisture distribution during the soaking and cooking of black rice were monitored by low-field nuclear magnetic resonance (NMR). Sensory characteristics of black rice were comprehensively evaluated by a sensory test and instrumental measurements. Particularly, for volatile components determined by GC–MS, the smell score by sensory evaluation and the response value of electronic noses were correlated to screen predominant volatile flavor contributor in cooked black rice.

## 2. Materials and Methods

### 2.1. Materials

Yangxian black rice cultivar (Yanghei No. 3) was obtained from Yangxian Lekang Ecological Agriculture Development Co. (Hanzhong, China). The samples were harvested in October 2021 and whole grains of black rice were died to 13.80% moisture content. Whole grains of black rice (250 g) were milled on a rice polisher (JM3010W, Shenzhen Mifresh Techology Co., Shenzhen, China) to obtain the milled black rice with different milling degrees by adjusting milling time and speed (gear shifting in the rice polisher) [10]. Black rice bran as a by-product of the milling process was collected after passing through a sieve (sieve hole diameter = 1 mm) inside the rice polisher.

Milling degree (MD) was calculated as the percentage weight loss between the milled black rice and whole black rice according to the established method [5,7,10,18]:

(1) $$MD=\frac{1-M_{milled\;black\;rice}}{M_{whole\;black\;rice}}\times 100\%$$

Since the bran layer accounted for about 10% of the entire grain weight [19], the prepared black rice samples with different milling degrees ranging from 0% to 10% approximately represented the gradient change from whole black rice to refined black rice.

### 2.2. Morphology Observation

Transverse sections of cooked whole black rice and milled samples after freeze-drying were prepared by using a sharp blade. The surface and transverse sections were sputter coated with gold before observing under a Scanning Electron Microscope (JSM-6390LV, Jeol Inc., Tokyo, Japan) operating at an acceleration voltage of 15 kV and magnifications of 40× and 750×.

### 2.3. Cooking Quality of Black Rice

Gelatinization time of black rice with different milling degrees during cooking was measured according to the international standard (ISO 14864:1998 [20]). Cooking quality (water uptake ratios, expanded volume, starch–iodine blue value/solid substance weight) was determined according to the method previously described [21,22].

### 2.4. Low-Field NMR Transverse Relaxation (T_2_) and Magnetic Resonance Imaging (MRI) Measurements

The low-field NMR relaxation measurements were conducted with a low-field NMR analyzer (NMI20-015V-I, Shanghai electronic technology Co., Ltd., Shanghai, China). Approximately 1 g of black rice in different stages (untreated samples, soaked for 30 min, and cooked for 10 min, 20 min, 40 min) was placed in a cylindrical glass tube (15 mm diameter) and inserted into the NMR probe. The analyzer operated at 32 °C with a resonance frequency of 21 MHz. The transverse relaxation time (T_2_) was measured using the Carr–Purcell–Meiboom–Gill (CPMG) sequence with the following parameters: SW = 100 kHz; RG = 20 dB; NECH = 10,000; TE = 0.3 ms; NS = 4, TW = 2000 ms. Parameters relating to the T_2_ were presented as follows: the T_2b_, T_21_, and T_22_ were the relaxation components, while the P_2b_, P_21_, and P_22_ were the corresponding area fractions.

Magnetic resonance imaging (MRI) was measured according to the method of Shang et al. with slight modification [23]. MRI was obtained using MSE sequence with the following parameters: Axial (X–Z) (Offset slice: 0.0 mm, Slices: 3; Slice width: 3.0 mm; Slice gap: 2.0 mm); FOV Read = 256 mm; FOV Phase = 192 mm; Averages = 2; TR = 500 ms; TE = 20 ms.

### 2.5. Eating Quality of Black Rice

#### 2.5.1. Cooking and Taste Analyzer Evaluation

A total of 30.00 g black rice with different milling degrees (A–F) was firstly washed 3–4 times and soaked for 30 min. The rice, at a rice to water ratio of 1:1.4 (*w*/*w*), was cooked for 40 min and insulated for 10 min.

Taste analyzer (STA1B, SATAKE Co., Ltd, Osaka, Japan) was used to assess the taste score and texture features of black rice grains. Briefly, the cooked black rice was firstly placed in the cooling device to cool for 20 min after gently flipping up. Then, 7.00 g of cooked black rice with different milling degrees (A–F) was accurately weighed and placed in a stainless-steel sample ring (height = 9 mm, diameter = 30 mm). The samples were then pressed into a pie using a specialized tool at room temperature. The prepared black rice samples were tested on the eating meter within the device for 1 min to obtain the values of taste score, appearance, and tastiness. Each side of the black rice sample was evaluated once, resulting in two measurements obtained per sample [24]. The transmitted light wavelength was set to 540 and 640 nm, and the reflected light wavelength was set to 540 and 970 nm [3]. The texture features (including hardness, stickiness, elasticity) of the cooked black rice were then measured using hardness and stickiness meter in the taste analyzer.

#### 2.5.2. Sensory Test

A sensory test is the fundamental method to reflect the eating quality of cooked rice. The sensory evaluation of rice cooking and eating quality was conducted according to the Chinese National Standard method GB/T 15682-2008 [25] with slight modification [7]. The cooked black rice with different milling degrees (A–F) was prepared by the method described in Section 2.5.1. The hot cooked black rice sample (10 g) was then placed on a plastic bowl covered with a lid and presented to panelists. Black rice samples were coded from 1 to 18 and the serving orders were randomized. Then, 25 trained members including 6 male and 19 female were selected to taste the cooked black rice and rate it based on primary descriptive attributes (including smell, appearance, texture, taste, cold rice texture). The attribute of appearance encompassed color, gloss of rice, and rice integrity as secondary attributes, while the attribute of texture included adhesiveness, springiness, hardness as secondary attributes (Table S1). Each attribute of black rice samples was scored in the corresponding description, and the overall sensory evaluation was determined by summing the score of primary descriptive attributes. The sensory test was conducted 2 h after the meal and the assessors were asked to rinse their mouths with water before each evaluation. The average value was calculated according to the results of scores provided by assessors.

### 2.6. Volatile Flavor Evaluation of Black Rice

#### 2.6.1. Electronic Noses

The electronic nose used was a FOX 4000 electronic nose (Alpha-M.O.S., Toulouse, France) equipped with 17 semi-conductor sensors. Electronic noses were measured according to the method of Huo et al. with slight modification [26]. The air condition unit was supplied with synthetic air and air flux was set to 150 mL/min. The cooked black rice sample (5 g) was placed in 20 mL gas vials. The vials were heated to 70 °C by the auto-sampler (incubation time = 300 s; agitation speed = 250 r/min). Then, 2.5 mL of the sample headspace was injected at 80 °C and the injection speed was 2.5 mL/s. The acquisition time and delay time were 120 s and 300 s, respectively.

#### 2.6.2. Gas Chromatography-Mass Spectrometry (GC-MS)

##### HS-SPME/GC-MS Analysis

First, 5 g of the cooked black rice was transferred into 20 mL glass headspace vial. The internal standards (methyl octanoate) were dissolved in HPLC-grade ethanol to prepare a solution of 6.7 mg/100 mL. A 0.5 μL of the internal standard solution was added to cooked rice and final concentration was 6.7 ng/g. A 2 cm long DVB/CAR/PDMS fiber (Supelco, Bellefonte, PA, USA) was used for HS-SPME and HS-SPME sampling was performed for 30 min at 60 °C. The SPME fiber was injected into the GC inlet and remained in the inlet for 5 min following extraction.

Volatiles were detected on a gas chromatography apparatus (GC7890A, Agilent Technologies, Santa Clara, CA, USA) coupled to a mass selective detector (MS 5975, Agilent Technologies, Santa Clara, CA, USA). An HP-5MS column (30 m × 0.25 mm × 0.25 µm) was used to separate volatile compounds at 50 °C for 2 min, increasing the temperature by 5 °C/min to a final temperature of 240 °C, followed by a 5 min hold at 240 °C. The flow rate was 1.0 mL/min of He (99.999%).

The electron impact ionization source temperature, quadrupole temperature, and transfer line temperature were set to 230 °C, 150 °C, and 280 °C, respectively. The electron energy was 70 eV. Volatiles were scanned over the range of *m*/*z* 30–350.

##### Identification and Relative Quantification of Volatiles

Volatiles were identified by comparing the retention times and mass spectra with those of authentic standard compounds. Volatiles without authentic standards were tentatively identified by searching NIST mass spectral libraries with <80% match score used as a cut off [27].

Relative concentration (ng/g) of volatiles was quantified based on an internal standard method. An *m*/*z* of 74 was used for area measurement of the internal standard. The specific extracted ion peak area of each compound was divided by the peak area of the internal standard. The area ratio was converted to relative concentration of the appropriate internal standard by multiplying by 6.70 ng/g black rice.

(2) $$\mathrm{Relative}\;\mathrm{concentration}\;(\frac{ng}{g})=\frac{\mathrm{Extracted}\;\mathrm{ion}\;\mathrm{peak}\;\mathrm{area}}{\mathrm{Extracted}\;\mathrm{ion}\;\mathrm{peak}\;\mathrm{area}\;\mathrm{of}\;\mathrm{internal}\;\mathrm{standard}}(\frac{6.70\;ng}{g})$$

### 2.7. Statistical Analysis

All experiments were performed at least in triplicate and the results were reported as means ± standard deviations (SD). Analysis of variance (ANOVA) of the data was performed, and a Fisher’s Least Significant Difference (LSD) test (confidence interval: 95%) was used to compare means of the different groups. For volatile profiling, all identified compounds were subjected to partial least squares discriminant analysis (PLS-DA), and those with variable importance in project VIP > 1 were regarded as differential volatile odors with important contributions to PLS-DA modeling, respectively. Pearson correlation cluster analysis was conducted to visualize the key volatile and sensors clustering that affected the perception of the smell of cooked black rice (Pearson correlation analysis was performed to determine the relationship between different variables at * *p* < 0.05 and ** *p* < 0.01).

## 3. Results and Discussion

### 3.1. Appearance and Microstructure of Cooked Black Rice

**Figure 1:**
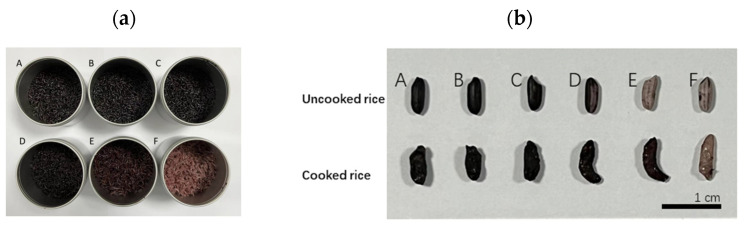

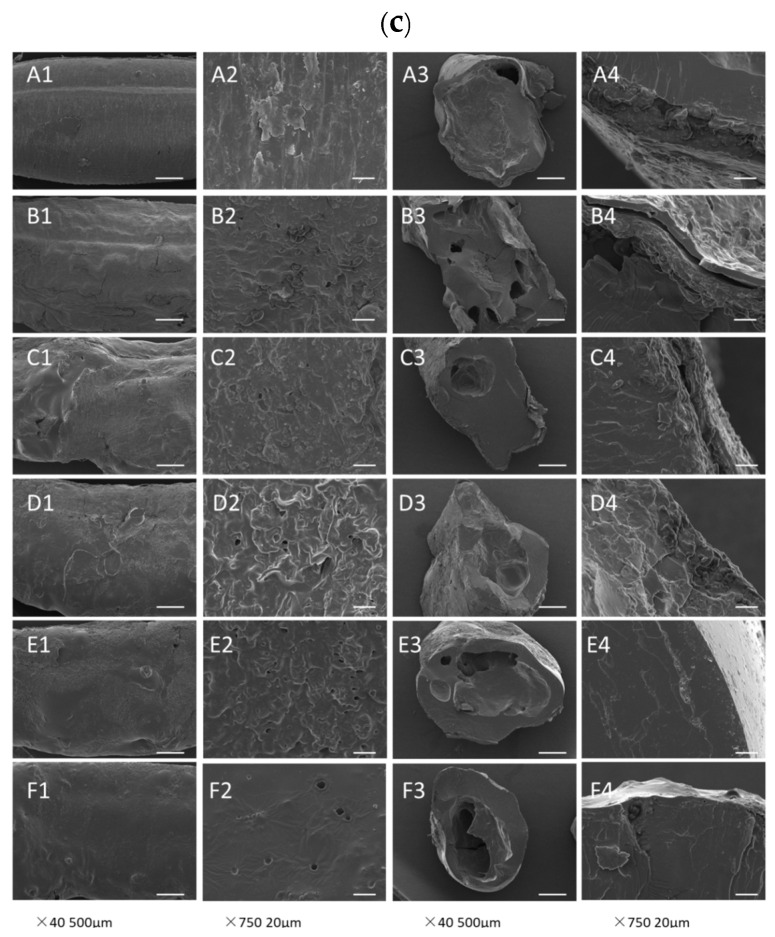
Appearance of cooked black rice samples with different milling degrees (A: 0%, B: 0.57%, C: 1.27%, D: 4.16%, E: 6.97%, F: 10.19%) (**a**); Comparison of uncooked and cooked rice (**b**); Scanning electron microscopy (SEM) pictures (A(1–4)–F(1–4)) of cooked black rice sample. Surface section: A1–F1 (scale bar: 500 μm) and A2–F2 (scale bar: 20 μm), transverse section: A3–F3 (scale bar: 500 μm) and A4–F4 (scale bar: 20 μm) (**c**).

The Yangxian black rice is classified as a medium-grain rice, with a length breadth ratio (L:B) of 2.1 to 3.0 (Figure 1a,b). The milling degree for each sample (A–F) was calculated as 0.00% (A), 0.57% ± 0.02% (B), 1.27% ± 0.02% (C), 4.16% ± 0.04% (D), 6.97% ± 0.14% (E), and 10.19% ± 0.10% (F). Given that the bran layer accounted for about 10% of the entire grain weight [19], the refined black rice (F) was approximately considered as having the bran layer removed. Since the pigment is concentrated in the outer bran layer, the color of cooked black rice gradually fades with increasing milling degree. The structure of cooked black rice samples was observed through SEM (Figure 1c). Distinct morphological differences in the integrity of surface and transverse sections of rice kernels were observed in black rice samples subjected to varying milling degrees. The surface and transverse sections revealed that the outer bran layer progressively flakes off and the inner endosperm is gradually exposed with the increasing milling degree. The appearance of holes in transverse sections was caused by starch gelatinization during cooking [28,29,30].

### 3.2. Cooking Quality of Black Rice

As shown in Table 1, the gelatinization time (optimal cooking time) gradually decreased with increasing milling degrees, indicating that the destruction of the bran layer shortened the cooking time. This result was consistent with the decreasing trend of gelatinization peak temperature of Differential Scanning Calorimetry (DSC) in a previous publication [10]. In addition, the value and trend of water uptake ratios and expanded volume were similar. The water uptake ratio of whole black rice (A) was significantly lower than those of refined grains (E, F). It was found that fat and wax in bran layers hindered the water uptake into the kernels for a given cooking duration since the hydrophobic waxy cuticle at the outer surface of the whole grain could offer a physical barrier [3,31,32]. The insufficient water absorption led to incomplete gelatinization and swelling of the starch in the central part of the grain, resulting in cooked whole black rice with a hard texture. Therefore, the slow gelatinization rate of the starch in whole black rice could be due to the limited water transfer at the kernel surface. The expanded volume and starch–iodine blue value per solid substance weight showed no discernible difference among samples of the same cultivar with different milling degrees.

### 3.3. Low-Field NMR Spin–Spin Relaxation (T_2_) Measurement

**Figure 2:**
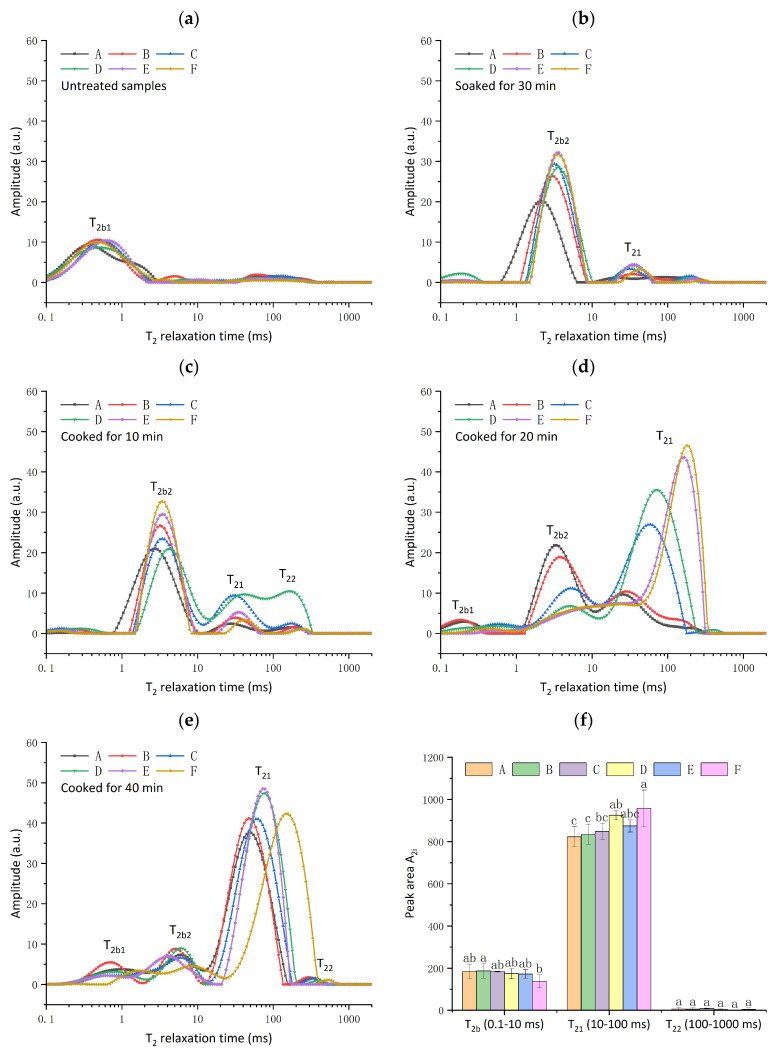
Distributed T_2_ relaxation time of black rice with different milling degrees (A: 0%, B: 0.57%, C: 1.27%, D: 4.16%, E: 6.97%, F: 10.19%) under different stages (untreated samples (**a**), soaked for 30 min (**b**), cooked for 10 min (**c**), 20 min (**d**), 40 min (**e**)), and peak area A_2i_ of black rice cooked for 40 min (**f**). Different superscript letters in the bar graph represented statistically significant differences (*p* < 0.05).

Low-field NMR is a non-destructive technique for testing the mobility and proportion of different fractions of water molecules. The T_2b_ (T_2b1_: 0.1–1 ms, T_2b2_: 1–10 ms,) component corresponds to water closely associated with macromolecules (tightly bound water), the T_21_ (10–100 ms) component reflects weakly bound water, and T_22_ (100–1000 ms) represents the free water [33]. Figure 2 shows the distributions of T_2_ relaxation times of black rice during the soaking and cooking stages. As shown in Figure 2a, the signal amplitude of the bound water (T_2b1_) in untreated samples occupied the major proportion since the black rice is a dried matter with little proton signal present. Soaking in excess water is an essential preliminary step to rice cooking. During the soaking process, the water diffused into black rice due to the moisture gradient between the surface and the center of the grain [34]. After soaking for 30 min, the bound water (T_2b2_) occupied the dominant portion of the grain and the immobilized water (T_21_) occupied a small proportion, indicating the water absorption process. A longer T_2_ corresponds to more molecular degrees of freedom [35]. Milled black rice displayed longer T_2b2_ values than whole black rice, suggesting that bran destruction facilitates water diffusion and absorption through crevices and chalky areas during soaking. During the cooking process, water is absorbed into the rice grains and hydrogen bonds between the starch molecules are gradually replaced by bonds between the starch and water molecules [36]. Water diffuses slowly from outside to inside the rice due to the crystalline organization of starch during cooking [36]. With the increase in cooking time, the T_2_ relaxation times continuously increased, indicating water absorption into the black rice grain. After 40 min of cooking, weakly bound water (T_21_) prevailed, and its peak value increased as the milling degree increased (Figure 2f). This suggested that the presence of bran hindered water absorption in the black rice starch.

**Figure 3:**
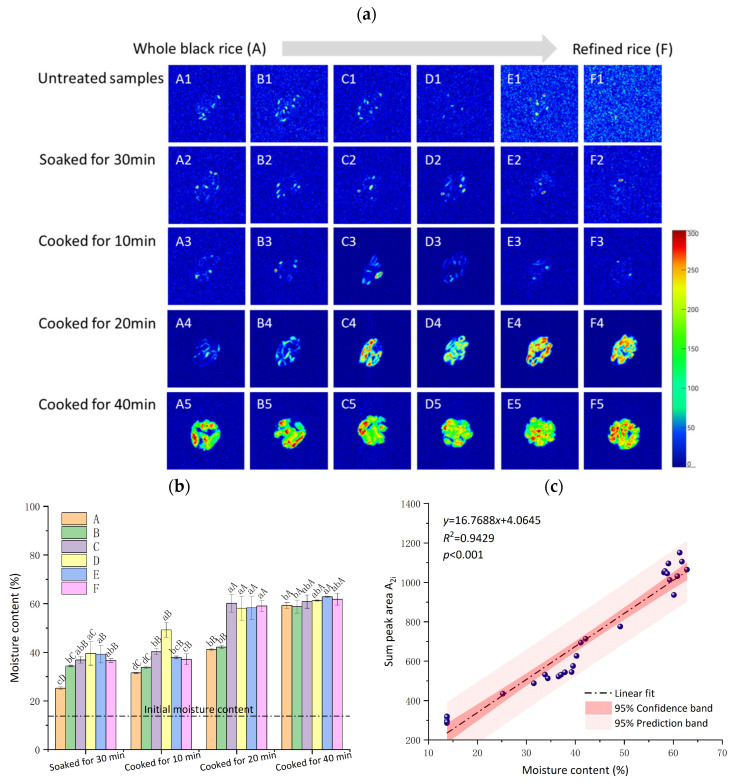
Changes in water distribution (**a**) of black rice with different milling degrees (A: 0%, B: 0.57%, C: 1.27%, D: 4.16%, E: 6.97%, F: 10.19%) under different stages (untreated samples (A1–F1), soaked for 30 min (A2–F2), and cooked for 10 min (A3–F3), 20 min (A4–F4), 40 min (A5–F5)), moisture content (**b**), correlation analysis of moisture content and sum peak area A_2i_ (**c**). In the bar graph, different superscript lower case letters in the bar graph indicated statistically significant differences between different black rice groups at the same soaked or cooked periods (*p* < 0.05), while different superscript upper case letters indicated statistically significant differences between different soaked or cooked periods in the same black rice group (*p* < 0.05).

The proton-weighted images of the water content of black rice with different cooking stages obtained by magnetic resonance imaging (MRI) are shown in Figure 3a. The proton-weighted images reveal the distribution of flow dynamic hydrogen protons, and the proton’s density is interpreted as the number of hydrogen protons. The pseudo-color image’s color scale from 0 to 300 corresponds to the color from blue to red, indicating that the fluidity of water increases gradually. The internal water distribution of black rice was obviously affected by both milling treatment and cooking time. The fluidity of water inside black rice increased with increasing cooking time. The fluidity of water inside C–F samples was greater than that of A and B after cooking for 20 min, which was due to differences in the integrity of black rice seeds. The distribution of red areas in the pseudo-color image of black rice gradually increased with the increase in milling degree after cooking for 40 min, indicating that the fluidity of water in various phases inside the rice grains was enhanced by milling treatment.

The distribution of hydrogen protons was associated with the water content of black rice in different cooking stages. The initial moisture content of all black rice samples was approximately 13.80%. After 30 min of soaking, the moisture content increased to between 25.20% and 39.54%. The moisture content of whole grain black rice A was significantly lower than that of the milled sample, indicating that the integrity of black rice prevented the water penetration during soaking. The gelatinization of the starch occurred and the water content increased from around 15% to 65% during the rice cooking [36]. The moisture content of all the samples showed an increasing trend with the extension of cooking time. The moisture contents of the milled samples (C–F) were significantly higher than those of samples (A and B) after cooking for 20 min, indicating easier cooking properties for milled samples (C–F). After cooking for 40 min, the water content of all samples reached basically the same level, ranging from 59.29% to 62.80%, indicating complete cooking and maximum possible grain water absorption. Correlation analysis showed a positive linear correlation (R^2^ = 0.9429) between water content and low-field NMR parameters (sum peak area). This fully demonstrated the quantitative reliability of low-field NMR data in measuring water concentration and distribution during cooking as all the water was detected [36,37].

### 3.4. Eating Quality of Black Rice

#### 3.4.1. Taste Analyzer

Rice taste analyzers developed in Japan operate on the basis of correlations between near-infrared reflectance measurements of the crucial constituents and the “taste” scores of the cooked rice [7,38]. It has been reported in the literature that the taste value of cooked rice is closely related to the physical and chemical parameters of rice [7,39]. The principle of the rice taste analyzers is based on the correction between near-infrared spectroscopy measurements of key constituents (such as amylose, protein, fatty acids, and moisture) and Japanese preferred sensory scores [38]. The “taste” scores assigned by the rice taste analyzer were designed to predict the comprehensive taste quality value [24]. As shown in Table 2, the taste score was significantly influenced by milling treatment in black rice cultivars. The taste value of cooked black rice increased first and then decreased with processing from whole black rice (A) to refined rice (F). Water diffusion and starch leaching are important factors affecting cooked rice quality [34]. Slightly milled black rice (B) and refined black rice (F) showed the highest and lowest taste value, respectively. This indicated that a moderate milling process can destroy the bran layer, thus improving the cooking quality. However, excessive milling and exposure of the endosperm lead to water absorption and dissolution during cooking, thus losing the proper viscoelasticity of black rice. In addition, there were significant differences in the quality traits of cooked rice (appearance, tastiness, hardness, and stickiness) among the black rice with different milling degrees. However, no differences were observed in terms of elasticity.

#### 3.4.2. Sensory Test of Eating Quality

A descriptive sensory test is generally carried out by trained panelists for measuring the intensity of the sensory attributes of cooked rice [16,17,40,41,42,43,44]. According to Chinese National Standard method GB/T 15682-2008, sensory evaluation eating qualities include primary descriptive attributes (smell, appearance, texture, taste, cold rice texture). The attribute of appearance encompassed color, gloss of rice, and rice integrity as secondary attributes, while the attribute of texture included adhesiveness, springiness, and hardness as secondary attributes (Table S1) [7]. The result indicated that milling treatment affected eating quality and scores for all sensory attributes (Table 3). The scores of the smell and appearance attributes decreased, while taste and cold rice texture increased with the increase in the milling degree. The milling treatment significantly reduced the smell score of cooked black rice in the sensory test since the volatile flavor compounds of rice were mainly distributed in the bran layer [45]. The consumers’ acceptance of naturally derived colorants was closely related to the image of health quality and food products [46]. The color of cooked rice was whitened by increasing the milling degree, which significantly lowered the sensory property score of appearance. Compared to whole grains, the texture feature (including adhesiveness, springiness, hardness) of milled black rice samples significantly improved. Specifically, the secondary attributes of appearance decreased while the secondary attributes of texture increased after milling treatment. The taste analyzer has been shown to be effective in indicating the taste quality of *japonica* milled rice [47]. However, the ability of the taste analyzer to accurately predict the sensory outcomes of cooked black rice remains uncertain, owing to the differing morphological and physicochemical properties between *japonica* milled rice and pigmented rice, as well as the complex processes involved in the mastication in the oral cavity. Partially, the slightly milled black rice (B) exhibited the highest overall score by the sensory test, which was consistent with the taste score trend from the taste analyzer. It indicated that the taste analyzer based on the multiple regression analysis of near-infrared spectroscopy data showed the potential to partially replace sensory evaluation in the study of cooked black rice. Despite the fact that the taste analyzer is a time-saving and labor-saving test for rice taste quality, this analyzer fails to describe the volatile flavor compounds information of cooked rice.

### 3.5. Volatile Flavor Evaluation of Black Rice

Flavor in rice (consisting of aroma and taste) is one of the most important properties affecting consumers’ preference [42,48]. Generally, there are three basic techniques available to assess the volatile flavor profile: (1) sensory analysis by a panel of trained experts, (2) gas chromatography-mass spectrometry (GC-MS) coupling to identify the volatile compounds, and (3) electronic noses [42,45,49].

#### 3.5.1. Electronic Noses

**Figure 4:**
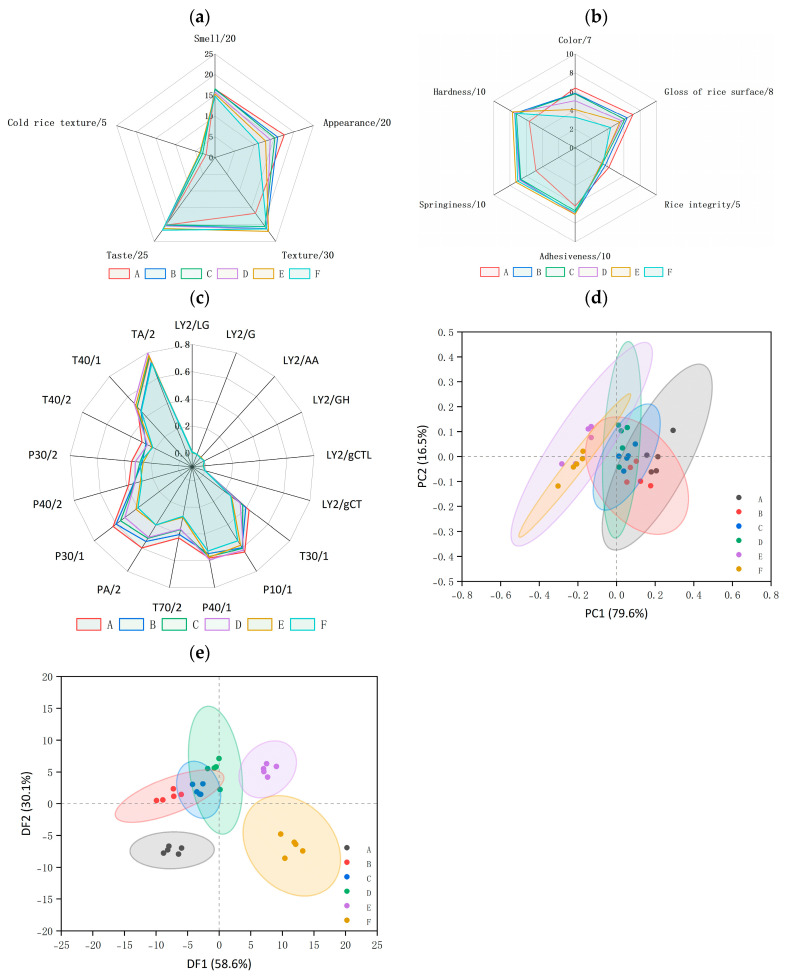
Radar map of primary descriptive attributes (**a**) (smell, appearance, texture, taste, cold rice texture) and secondary attributes (**b**) of appearance (including color, gloss of rice, rice integrity) and texture (including adhesiveness, springiness, hardness) of black rice with different milling degrees; Radar map of response value of 17 sensors (**c**); Principal component score plot (**d**) and corresponding discriminant function score plot (**e**).

The aroma of rice is one of most important factors in determining quality. The electronic nose (E-nose) is a system designed to mimic human olfaction, relying on an array of sensors and appropriate pattern recognition methods [45]. This instrument is capable of detecting subtle changes in sample composition and is primarily used for rice classification and predicting the degree of difference between test samples [45]. Response values of the electronic nose towards the black rice with different milling degrees are listed in Table 4 and Figure 4c. The response values of the sensors (T30/1, T70/2, PA/2, P30/1, P40/2, and T40/2) decreased as the milling degree increased. The response value of the sensors (P10/1, P40/1, P30/2, T40/1, and TA/2) showed a trend of decreasing, increasing, and then decreasing with increased milling degree. The aroma attributes of cooked rice were reported to greatly correlate with certain volatile compounds, such as aldehydes and heterocycles, and volatile flavor compounds varied with milling degree [41,43]. As shown in Table 4, PA/2, P30/1, P30/2, and TA/2 are sensitive to organic compounds; P40/1, P40/2, T40/2, and T40/1 are sensitive to gases with high oxidizing power; T30/1 is sensitive to polar compounds; P10/1 is sensitive to nonpolar compounds (including hydrocarbon, ammonia, chlorine); and T70/2 is sensitive to aromatic compounds (such as toluene and xylene) [50,51]. The difference response values of sensors indicated that varied volatile flavor compounds exist in cooked black rice with different milling degrees [39].

The PCA analysis, displayed in Figure 4d, showed that the importance of principal component 1 (PC1) and component 2 (PC2) were 79.6% and 16.5%, respectively. The cumulative importance of PC1 and PC2 was 96.1%, indicating the effectiveness of PCA in reflecting most of the original sample’s odor data. The difference between whole black rice and milled black rice samples gradually increased with the increase in milling degree. The confidence ellipses of whole black rice (A) and refined black rice (E and F) did not overlap, indicating significant difference in the odor characteristics. Discriminant factor analysis (DFA) was applied to further provide precise discrimination between groups. In DFA analysis, the importance of discriminant factor 1 (DF1) and factor 2 (DF2) were 58.6% and 30.1%, respectively. A cumulative importance of DF1 and DF2 was 88.7%. DFA results (Figure 4e) showed A, E and F were grouped in lower left, upper right, lower right quadrants, respectively. The milled samples (B, C, and D) overlapped in the upper left quadrant, indicating that these samples may have similar volatile profiles in the headspace emitted. These results showed that both PCA and DFA can be effectively used to separate/discriminate odor characteristics of black rice samples, with DFA exhibiting more effective discriminating results.

#### 3.5.2. GC-MS

Twelve volatile compounds, including one aliphatic/alicyclic ketones, three aliphatic aldehydes, three aromatics, one aliphatic alcohols, and two alkanes, and two N-containing compounds were identified in cooked black rice by HS-SPME with GC-MS (Table 5 and Table 6). Among them, cooked whole grain black rice (A) contained a significantly larger amount of hexanal, p-xylene, 2-pentyl-furan, and guaiacol than refined black rice (F). This indicated these volatile compounds were mainly distributed in the bran and easily removed by milling. The aldehydes, products derived predominantly via lipid oxidation and decomposition, had a low odor threshold. Aliphatic aldehydes (including hexanal, nonanal, decanal) have been identified and are considered as odor-active compounds in various ratios of cooked black and white rice [39,49,52,53,54]. Hexanal and 1-octen-3-ol are reported to contribute greatly to the aroma attribute of cooked rice [39,49]. 1-Octen-3-ol and 2-amino-5-methylbenzoic acid are identified as volatile odors of raw and processed black rice [55]. Guaiacol and p-xylene are representative volatile compounds of black rice [52]. Guaiacol is responsible for a smoky flavor, a characteristic volatile flavor of black rice with a low odor threshold (3 ppb) [56]. The present study found that the guaiacol released from black rice showed an increasing (14.54–19.39 ng/g) and then decreasing (19.39–5.51 ng/g) trend with increasing degree of milling, indicating that moderate milling favors the release of guaiacol aroma substances, while excessive milling decreases the content of guaiacol. The increased trend of guaiacol by moderate milling may due to the fact that the fragmentation occurring in this process facilitates the release of typical rice-related aroma components [57]. 2-Pentyl-furan is an oxidation product of linoleic acid and linolenic acid and contributes floral and fruit odor with a 6 ppb threshold value [27,48,49,58]. The content of 2-pentyl-furan decreased with increasing milling degree (54.84–12.72 ng/g), consistent with trends in previous reports [56].

The odor activity value (OAV) refers to the ratio of the volatile concentration in the aroma system to its odor threshold. OAV is an indicator of the sensory impact of aroma compound on the overall aroma [59]. A higher OAV value suggests a greater contribution of the component to the overall aroma. Generally, aroma components with OAV > 1 are considered as modified aroma components, while aroma components with OAV > 10 are named key aroma components [60]. Hexanal, nonanal, decanal, and 1-octen-3-ol were considered key aroma components, while p-xylene, 2-pentyl-furan, and 2-methoxy-phenol were modified aroma components in cooked whole black rice (Table 6).

**Figure 5:**
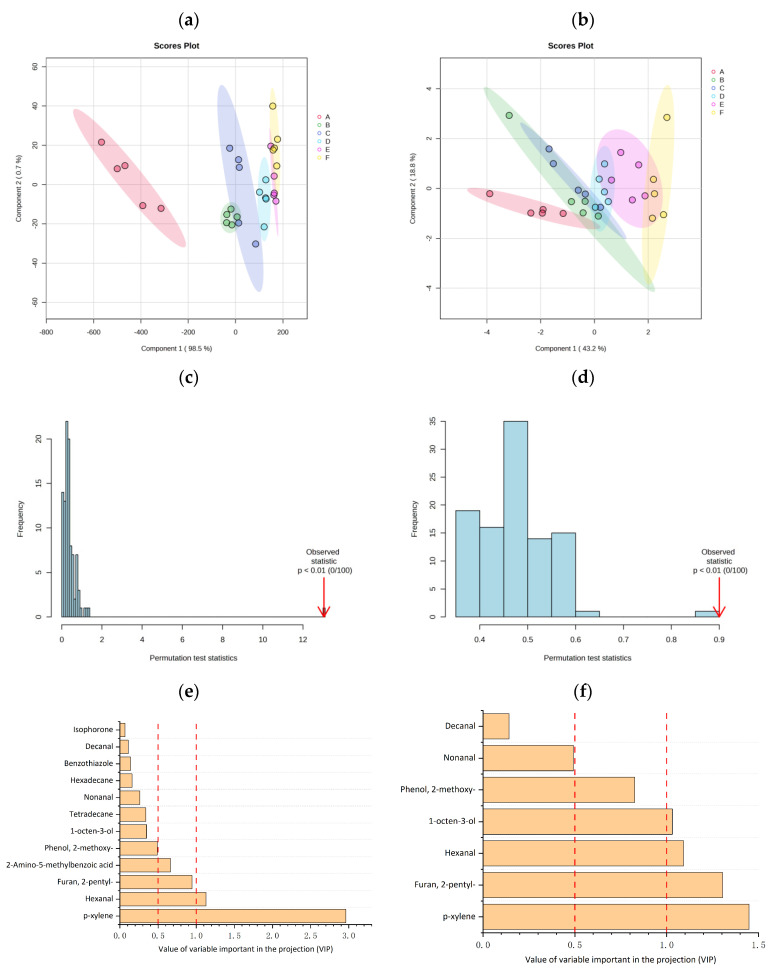
PLS-DA score plot based on volatiles content (**a**) and OAV (**b**), permutation test by PLS-DA based on volatile odor content (**c**) and OAV (**d**), value of variable importance in the projection (VIP) of variables by PLS-DA of volatiles identified in cooked black rice samples based on volatile odor content (**e**) and OAV (**f**).

A supervised model PLS-DA was then conducted to optimize sample separation and identify volatiles’ differences (relative concentration or OAV) arising from milling degree [61]. In the analysis, the milling degree was set as classes and the model was validated by an internal validation method. For the latter, validation parameters (R^2^ and Q^2^) indicated the quality of the model. R^2^ and Q^2^ specified the proportion of variation in the data that is explained and predicted by the model, respectively. The validation analysis of the PLS-DA model based on relative concentration and OAV yielded R^2^ of 0.9497 and 0.9208, and Q^2^ of 0.9174 and 0.8098, respectively. This demonstrated good explanatory and predictive capabilities. A permutation test (100 permutations) was performed to assess the risk of overfitting. A *p*-value below 0.01 in Figure 5c,d indicated that the PLS-DA model is validated. The contribution of volatiles was evaluated using variable importance in projection (VIP) plots and VIP values > 1.00 suggested a significant influence on the PLS model [61]. Hexanal and p-xylene were the top-ranked volatiles in VIP plots based on relative concentration, while p-xylene, 2-pentyl-furan, and hexanal were the top-ranked volatiles in VIP plots based on OAV (VIP > 1.00) (Figure 5e).

#### 3.5.3. Pearson Correlation Cluster Analysis

**Figure 6:**
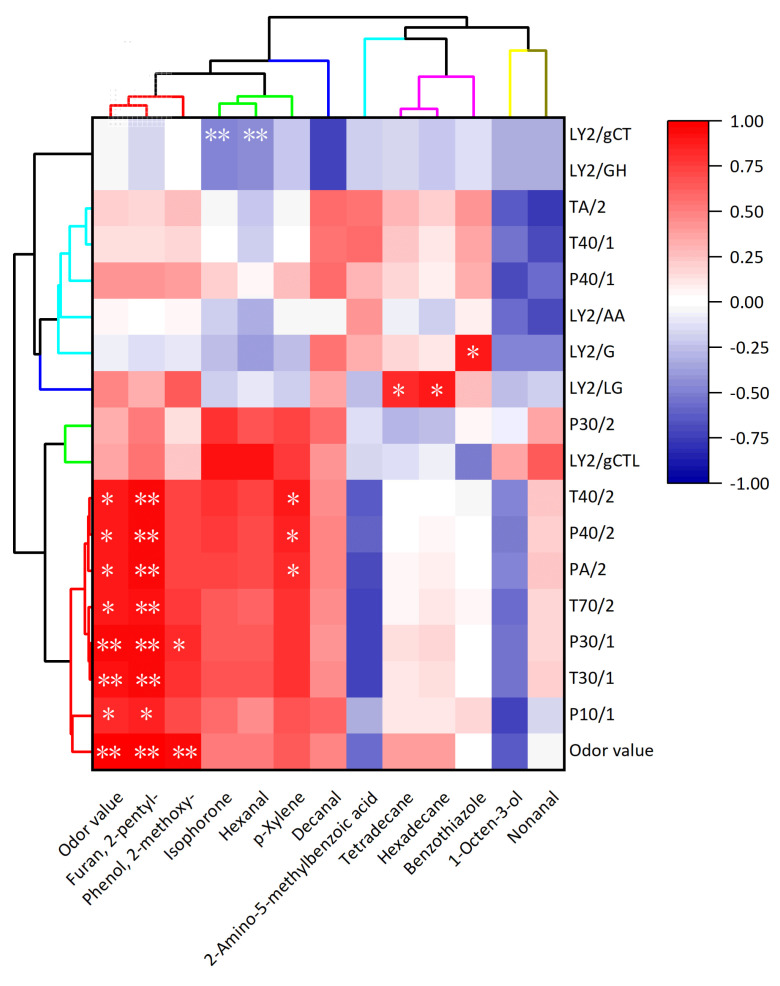
Heat map of hierarchical cluster analysis of Pearson correlation coefficient between odor value (smell score in sensory test) and response value of sensors in electronic noses or relative concentration of volatiles (* *p* < 0.05; ** *p* < 0.01).

With a theoretical odor detection limit of approximately 10^−19^ mol [62], the human nose makes sensory evaluation a valuable and highly sensitive approach for analyzing rice aroma and odor-active volatiles [45]. Therefore, the Pearson correlation coefficient was calculated to visualize the key volatiles and sensors clustering that affect the perception of cooked black rice aroma (smell score in sensory test) in Pearson correlation cluster analysis. Cluster analysis was performed in order to determine the key volatiles and sensors of electronic noses reflecting odor value (smell score in sensory test). According to the Pearson correlation coefficient, 12 volatile compounds were grouped into 7 categories and 17 sensors were grouped into 5 categories (Figure 6). On the X-axis, the odor value (smell score in sensory test) exhibited a significant positive correlation with the concentration of 2-pentyl-furan and 2-methoxy-phenol (guaiacol). Additionally, cluster analysis classified these volatile compounds and odor values (smell score in sensory test) in the same category, indicating that the trend characteristics of these substances were similar to the smell score in sensory test. Therefore, the changes in the odor value (smell score in sensory test) of black rice with different milling degrees were mainly caused by the changes in the content of 2-pentyl-furan and guaiacol. Many researchers have studied volatile compounds in cooked black rice and found that guaiacol presented in black rice bran was responsible for the characteristic component in black rice [63]. It was reported that the aroma difference between red and black rice bran was caused by the content difference of guaiacol [63]. Guaiacol was also reported to be a principal contributor to the unique aroma of cooked black rice [52]. Similarly, 2-pentyl-furan was recognized as a characteristic odor-active compound in California long-grain rice [64].

The electronic nose (e-nose), as the simulator of the human sense of smell, is highly sensitive to odor information, allowing it to detect subtle variations in volatile compounds that result in distinct sensor responses [65]. On the Y-axis, the odor value (smell score in sensory test) showed a significant positive correlation with sensors (T40/2, P40/2, PA/2, T70/2, P30/1, T30/1, and P10/1). Moreover, cluster analysis also classified these sensors and odor values (smell score in sensory test) in the same category, suggesting that the trend characteristics in these sensors were similar to those in the odor value (smell score in sensory test). Therefore, the response values of the sensors (T40/2, P40/2, PA/2, T70/2, P30/1, T30/1, and P10/1) were able to distinguish the sensory odor characteristics in the real population since the sensors reacted differently to the volatile compounds.

## 4. Conclusions

This study examined the cooking and sensory properties of Chinese black rice subjected to gradient milling. The findings revealed a significant decrease in gelatinization time with increasing milling degrees, while the water uptake ratio of whole black rice was notably lower than that of refined grains. Furthermore, low-field NMR was employed to assess water concentration and the distribution of black rice during soaking and cooking. The results suggested that bran layers of black rice acted as a physical barrier, impeding the water’s penetration into the kernels during both soaking and cooking. This difference in water penetration and distribution significantly impacted the eating quality of cooked rice. Furthermore, sensory attributes influencing the texture features of cooked black rice were closely linked to the milling treatment. The slightly milled black rice (B, milling degree: 0.57%) showed the highest scores for all sensory attributes both in the sensory test and taste analyzer measurement. Furthermore, the changes in the odor value (smell score in sensory test) in the sensory test of black rice with different milling degrees were mainly caused by the changes in the content of 2-pentyl-furan and guaiacol. To retain the bioactive phytochemicals and volatile flavor compounds in the bran layer, it is recommended to avoid excessive milling of black rice. This finding supported the observation that moderately milled black rice significantly enhanced the taste characteristics of whole black rice while preserving the maximum amount of bran fraction, making it a viable alternative staple food. Overall, this work not only contributed to our understanding of the interplay between milling, cooking, and the sensory aspects of black rice, but also offered valuable insights into the moderate milling of black rice to balance taste and nutrition.

In the present study, trained panelists were recruited as tasters for the cooked black rice with varying milling degrees, rather than untrained consumers. To assess the acceptability and palatability of cooked black rice, future research should incorporate a consumer acceptance test involving untrained individuals and evaluate whether the taste analyzer can accurately predict the acceptability of cooked black rice. Furthermore, it is crucial to compare the results across larger populations to distinguish the effects of demographic characteristics such as age, gender, region, and training experience on sensory scores to provide a more accurate sensory description of cooked black rice.

## Figures and Tables

**Table 1:** Cooking characteristics of black rice with different milling degrees (A–F).

Sample	Gelatinization Time (min)	Water Uptake Ratios (%)	Expanded Volume (%)	Starch–Iodine Blue Value/Solid Substance Weight
A	36.21 ± 2.15 ^a^	188.29 ± 4.77 ^b^	187.88 ± 5.25 ^b^	0.81 ± 0.26 ^a^
B	36.85 ± 1.20 ^a^	193.53 ± 1.79 ^a^	193.94 ± 5.25 ^ab^	0.72 ± 0.06 ^a^
C	32.78 ± 1.46 ^b^	194.57 ± 1.62 ^a^	196.97 ± 5.25 ^a^	0.84 ± 0.13 ^a^
D	26.15 ± 0.56 ^c^	192.52 ± 3.59 ^ab^	193.94 ± 5.25 ^ab^	0.66 ± 0.07 ^a^
E	23.54 ± 0.51 ^d^	194.05 ± 0.59 ^a^	190.91 ± 0.00 ^ab^	0.81 ± 0.21 ^a^
F	23.82 ± 0.74 ^d^	194.05 ± 1.86 ^a^	193.94 ± 5.25 ^ab^	0.82 ± 0.03 ^a^

A–F: Cooked black rice with different milling degrees (A: 0%, B: 0.57%, C: 1.27%, D: 4.16%, E: 6.97%, F: 10.19%). Different lowercase letters in the columns represented statistically significant differences (*p* < 0.05).

**Table 2:** Eating quality traits of cooked black rice with different milling degrees (A–F) evaluated by rice taste analyzer.

Samples	Taste Score/100	Appearance/10	Tastiness/10	Hardness/kgf	Stickiness/kgf	Elasticity
A	82.50 ± 0.84 ^c^	1.02 ± 0.23 ^c^	3.70 ± 0.24 ^c^	1.24 ± 0.18 ^ab^	0.13 ± 0.07 ^bc^	0.94 ± 0.02 ^a^
B	86.17 ± 0.41 ^a^	2.03 ± 0.16 ^a^	4.75 ± 0.15 ^a^	1.29 ± 0.13 ^ab^	0.18 ± 0.01 ^ab^	0.94 ± 0.03 ^a^
C	84.67 ± 0.52 ^b^	1.50 ± 0.11 ^b^	4.08 ± 0.17 ^b^	1.33 ± 0.13 ^a^	0.21 ± 0.06 ^a^	0.95 ± 0.02 ^a^
D	84.17 ± 0.41 ^b^	1.08 ± 0.13 ^c^	3.48 ± 0.13 ^d^	1.12 ± 0.08 ^b^	0.11 ± 0.00 ^cd^	0.96 ± 0.02 ^a^
E	80.83 ± 0.75 ^d^	0.20 ± 0.00 ^d^	1.92 ± 0.19 ^e^	0.74 ± 0.06 ^c^	0.06 ± 0.03 ^d^	0.95 ± 0.01 ^a^
F	78.00 ± 0.00 ^e^	0.20 ± 0.00 ^d^	0.80 ± 0.09 ^f^	0.63 ± 0.04 ^c^	0.09 ± 0.02 ^cd^	0.96 ± 0.02 ^a^

A–F: Cooked black rice with different milling degrees (A: 0%, B: 0.57%, C: 1.27%, D: 4.16%, E: 6.97%, F: 10.19%). Different lowercase letters in the columns represented statistically significant differences (*p* < 0.05).

**Table 3:** Sensory evaluation of eating quality of cooked black rice with different milling degrees (A–F).

Samples	Smell /20	Appearance ^1^ /20	Texture ^2^ /30	Taste /25	Cold Rice texture/5	Score for All Sensory Attributes ^3^/100
A	16.50 ± 1.93 ^ab^	17.62 ± 1.49 ^a^	16.64 ± 4.04 ^b^	20.12 ± 2.51 ^b^	2.26 ± 1.05 ^c^	73.14 ± 6.66 ^ab^
B	16.40 ± 1.53 ^ab^	15.92 ± 1.73 ^b^	21.20 ± 2.55 ^a^	20.18 ± 2.66 ^b^	3.14 ± 0.94 ^b^	76.84 ± 5.49 ^a^
C	16.58 ± 1.15 ^a^	15.22 ± 1.89 ^b^	20.58 ± 2.73 ^a^	20.06 ± 2.34 ^b^	3.14 ± 0.71 ^b^	75.58 ± 4.80 ^ab^
D	15.80 ± 1.72 ^abc^	14.02 ± 1.63 ^c^	21.32 ± 3.09 ^a^	20.42 ± 2.51 ^ab^	3.64 ± 0.84 ^a^	75.20 ± 6.20 ^ab^
E	15.36 ± 2.56 ^bc^	12.86 ± 2.22 ^c^	22.08 ± 3.28 ^a^	21.18 ± 2.61 ^ab^	3.86 ± 0.82 ^a^	75.34 ± 6.82 ^ab^
F	14.80 ± 3.17 ^c^	11.04 ± 3.21 ^d^	21.30 ± 4.47 ^a^	21.62 ± 2.51 ^a^	3.64 ± 0.95 ^a^	72.40 ± 9.10 ^b^

A–F: Cooked black rice with different milling degrees (A: 0%, B: 0.57%, C: 1.27%, D: 4.16%, E: 6.97%, F: 10.19%). ^1^: The attribute of appearance encompassed color, gloss of rice, rice integrity as secondary attributes. ^2^: The attribute of texture included adhesiveness, springiness, hardness as secondary attributes. ^3^: The score for all sensory attributes was assessed on a 100-point scale and primary attribute scores of smell, appearance, texture, taste, cold rice texture accounted for 20, 20, 30, 25, 5, respectively. Different lowercase letters in the columns represented statistically significant differences (*p* < 0.05).

**Table 4:** Response value of electronic noses towards the black rice with different milling degrees (A–F) (n = 5).

	**Samples**	Volatile Description	A	B	C	D	E	F
Sensors								
LY2/LG		Fluoride, chloride, oxynitride, sulphide	0.004 ± 0.005 ^a^	0.009 ± 0.011 ^a^	0.011 ± 0.009 ^a^	0.006 ± 0.006 ^a^	0.004 ± 0.004 ^a^	0.006 ± 0.006 ^a^
LY2/G		Ammonia, amines, carbon oxygen compounds	−0.001 ± 0.000 ^a^	−0.001 ± 0.000 ^a^	−0.001 ± 0.001 ^a^	−0.000 ± 0.000 ^a^	−0.001 ± 0.000 ^a^	−0.001 ± 0.001 ^a^
LY2/AA		Alcohol, acetone, ammonia	−0.007 ± 0.005 ^a^	−0.008 ± 0.005 ^a^	−0.008 ± 0.009 ^a^	−0.006 ± 0.005 ^a^	−0.004 ± 0.002 ^a^	−0.010 ± 0.006 ^a^
LY2/GH		Ammonia, amines compounds	−0.001 ± 0.000 ^a^	0.000 ± 0.000 ^a^	−0.001 ± 0.001 ^a^	−0.001 ± 0.001 ^a^	−0.000 ± 0.000 ^a^	−0.001 ± 0.001 ^a^
LY2/gCTL		Hydrogen sulfide	−0.013 ± 0.009 ^a^	−0.020 ± 0.011 ^a^	−0.016 ± 0.006 ^a^	−0.019 ± 0.007 ^a^	−0.020 ± 0.011 ^a^	−0.017 ± 0.007 ^a^
LY2/gCT		Propane, butane	−0.003 ± 0.001 ^c^	−0.002 ± 0.001 ^a^	−0.003 ± 0.001 ^abc^	−0.003 ± 0.001 ^bc^	−0.002 ± 0.001 ^ab^	−0.003 ± 0.001 ^abc^
T30/1		Polar compound, hydrogen chloride	0.424 ± 0.016 ^a^	0.394 ± 0.024 ^b^	0.366 ± 0.010 ^c^	0.343 ± 0.007 ^d^	0.264 ± 0.015 ^e^	0.256 ± 0.014 ^e^
P10/1		Nonpolar compound: hydrocarbon, ammonia, chlorine	0.634 ± 0.016 ^a^	0.597 ± 0.012 ^bc^	0.603 ± 0.014 ^bc^	0.618 ± 0.019 ^ab^	0.576 ± 0.033 ^c^	0.537 ± 0.024 ^d^
P40/1		Fluorine, chlorine	0.580 ± 0.025 ^a^	0.547 ± 0.015 ^bc^	0.569 ± 0.017 ^ab^	0.593 ± 0.025 ^a^	0.574 ± 0.032 ^ab^	0.528 ± 0.025 ^c^
T70/2		Toluene, xylene, carbon monoxide	0.430 ± 0.022 ^a^	0.405 ± 0.024 ^a^	0.368 ± 0.011 ^b^	0.364 ± 0.016 ^b^	0.278 ± 0.029 ^c^	0.270 ± 0.008 ^c^
PA/2		Ethanol, ammonia, amine compound	0.600 ± 0.024 ^a^	0.543 ± 0.020 ^b^	0.516 ± 0.020 ^bc^	0.507 ± 0.007 ^c^	0.403 ± 0.029 ^d^	0.403 ± 0.028 ^d^
P30/1		Hydrocarbons, ammonia, ethanol	0.626 ± 0.016 ^a^	0.598 ± 0.028 ^b^	0.558 ± 0.013 ^c^	0.518 ± 0.020 ^d^	0.415 ± 0.024 ^e^	0.397 ± 0.023 ^e^
P40/2		Chlorine, hydrogen sulfide, fluoride	0.384 ± 0.015 ^a^	0.346 ± 0.012 ^b^	0.340 ± 0.011 ^b^	0.337 ± 0.010 ^b^	0.294 ± 0.021 ^c^	0.283 ± 0.017 ^c^
P30/2		Hydrogen sulphide, ketone	0.345 ± 0.043 ^a^	0.267 ± 0.014 ^c^	0.283 ± 0.016 ^bc^	0.318 ± 0.032 ^ab^	0.260 ± 0.030 ^c^	0.282 ± 0.021 ^bc^
T40/2		Chlorine	0.310 ± 0.011 ^a^	0.278 ± 0.011 ^b^	0.270 ± 0.009 ^b^	0.265 ± 0.006 ^b^	0.229 ± 0.019 ^c^	0.222 ± 0.014 ^c^
T40/1		Fluorine	0.496 ± 0.046 ^ab^	0.457 ± 0.026 ^b^	0.497 ± 0.029 ^ab^	0.530 ± 0.040 ^a^	0.512 ± 0.044 ^a^	0.453 ± 0.032 ^b^
TA/2		Ethanol	0.760 ± 0.034 ^abc^	0.729 ± 0.027 ^bc^	0.768 ± 0.022 ^ab^	0.798 ± 0.037 ^a^	0.779 ± 0.046 ^a^	0.716 ± 0.035 ^c^

A–F: Cooked black rice with different milling degrees (A: 0%, B: 0.57%, C: 1.27%, D: 4.16%, E: 6.97%, F: 10.19%). Different lowercase letters in the lines represented statistically significant differences (*p* < 0.05).

**Table 5:** Relative concentration (ng/g) of volatiles identified in cooked black rice with different milling degrees (A–F).

Volatile Compound	CAS No.	A	B	C	D	E	F
Aliphatic/Alicyclic Ketones							
Isophorone	78-59-1	13.28 ± 2.17 ^a^	11.30 ± 0.84 ^ab^	11.79 ± 2.18 ^ab^	11.23 ± 1.81 ^ab^	10.92 ± 1.93 ^b^	11.39 ± 1.18 ^ab^
Aliphatic Aldehydes							
Hexanal	66-25-1	148.56 ± 32.75 ^a^	87.77 ± 5.53 ^bc^	101.70 ± 31.22 ^b^	70.09 ± 8.31 ^c^	67.25 ± 10.16 ^c^	90.93 ± 17.73 ^bc^
Nonanal	124-19-6	24.78 ± 5.13 ^a^	19.17 ± 3.19 ^b^	15.39 ± 7.04 ^a^	12.95 ± 1.27 ^c^	12.78 ± 3.09 ^c^	24.63 ± 2.56 ^a^
Decanal	112-31-2	3.50 ± 1.30 ^a^	2.34 ± 0.87 ^a^	4.50 ± 1.94 ^a^	4.41 ± 3.96 ^a^	2.06 ± 0.59 ^a^	2.48 ± 0.84 ^a^
Aromatics							
p-Xylene	106-42-3	655.40 ± 94.36 ^a^	230.86 ± 18.37 ^b^	188.26 ± 36.98 ^b^	91.57 ± 11.26 ^c^	51.86 ± 7.27 ^c^	45.15 ± 8.79 ^c^
Furan, 2-pentyl-	3777-69-3	54.84 ± 11.20 ^a^	41.72 ± 3.37 ^b^	47.93 ± 10.93 ^ab^	30.85 ± 5.40 ^c^	21.46 ± 4.16 ^cd^	12.72 ± 4.95 ^d^
Phenol, 2-methoxy- (Guaiacol)	90-05-1	14.54 ± 2.38 ^b^	15.69 ± 2.74 ^ab^	19.39 ± 4.79 ^a^	11.96 ± 3.40 ^bc^	9.92 ± 1.31 ^c^	5.51 ± 1.46 ^d^
Aliphatic Alcohols							
1-Octen-3-ol	3391-86-4	19.56 ± 4.07 ^b^	16.67 ± 2.25 ^b^	18.38 ± 5.14 ^b^	16.01 ± 3.03 ^b^	18.71 ± 3.70 ^b^	24.93 ± 2.23 ^a^
Alkanes							
Tetradecane	629-59-4	4.26 ± 1.54 ^d^	9.00 ± 2.27 ^bc^	17.60 ± 6.22 ^a^	10.74 ± 2.20 ^b^	7.50 ± 1.40 ^bcd^	6.47 ± 1.69 ^cd^
Hexadecane	544-76-3	3.34 ± 1.07 ^c^	5.62 ± 1.70 ^b^	9.47 ± 3.00 ^a^	6.10 ± 1.32 ^b^	4.24 ± 1.22 ^bc^	4.60 ± 1.20 ^bc^
N-Containing Compounds							
Benzothiazole	95-16-9	12.70 ± 6.19 ^b^	15.88 ± 2.55 ^ab^	15.10 ± 3.39 ^ab^	19.77 ± 3.85 ^a^	13.84 ± 3.88 ^b^	14.46 ± 1.90 ^b^
2-Amino-5-methylbenzoic acid	2941-78-8	44.07 ± 6.68 ^bc^	35.66 ± 5.62 ^c^	55.35 ± 16.23 ^ab^	58.26 ± 8.26 ^a^	62.62 ± 12.00 ^a^	55.10 ± 5.27 ^ab^

Values expressed as octanoic acid methyl ester equivalent (ng/g) and given as average ± standard deviation (n = 5). Different lowercase letters in the lines represented statistically significant differences (*p* < 0.05).

**Table 6:** Odor description, odor threshold, and OAV in cooked black rice with different milling degrees (A–F).

Compound	Odor Description	Odor Threshold (ppb)	OAV (A)	OAV (B)	OAV (C)	OAV (D)	OAV (E)	OAV (F)
Hexanal	Green tomato, green, grassy	4.5	33.01 ± 7.28 ^a^	19.50 ± 1.18 ^bc^	22.60 ± 6.94 ^b^	15.58 ± 1.85 ^c^	14.94 ± 2.26 ^c^	20.21 ± 3.94 ^bc^
Nonanal	Aldehydic, waxy, citrus, tart, sweet	1	24.78 ± 5.13 ^a^	19.17 ± 3.19 ^b^	15.39 ± 7.04 ^a^	12.95 ± 1.27 ^c^	12.78 ± 3.09 ^c^	24.63 ± 2.56 ^a^
Decanal	Citrus	0.1	35.00 ± 13.00 ^a^	23.40 ± 8.70 ^a^	45.00 ± 19.40 ^a^	44.1 ± 39.60 ^a^	20.60 ± 5.90 ^a^	24.80 ± 8.40 ^a^
p-Xylene	Medicinal	530	1.23 ± 0.18 ^a^	0.44 ± 0.03 ^b^	0.36 ± 0.07 ^b^	0.17 ± 0.02 ^c^	0.10 ± 0.01 ^c^	0.09 ± 0.02 ^c^
Furan, 2-pentyl-	Floral, fruit	6	9.14 ± 1.87 ^a^	6.95 ± 0.56 ^b^	7.99 ± 1.82 ^ab^	5.14 ± 0.90 ^c^	3.58 ± 0.69 ^cd^	2.12 ± 0.83 ^d^
Phenol, 2-methoxy-	Smoky, black rice-like	3	4.85 ± 0.79 ^b^	5.23 ± 0.91 ^ab^	6.46 ± 1.60 ^a^	3.99 ± 1.13 ^bc^	3.31 ± 0.44 ^c^	1.83 ± 0.49 ^d^
1-Octen-3-ol	Mushroom	1	19.56 ± 4.07 ^b^	16.67 ± 2.25 ^b^	18.38 ± 5.14 ^b^	16.01 ± 3.03 ^b^	18.71 ± 3.70 ^b^	24.93 ± 2.23 ^a^

Different lowercase letters in the lines represented statistically significant differences (*p* < 0.05).

## Data Availability

The original contributions presented in the study are included in the article/Supplementary Material, further inquiries can be directed to the corresponding author.

## Supplementary Materials

The following supporting information can be downloaded at: https://www.mdpi.com/article/10.3390/foods13213453/s1, Table S1: Detailed rating rules and record form for sensory evaluation of cooked black rice [25].
