# Supplementary material for: Influence of Gradient Milling on Cooking and Sensory Attributes of Chinese Black Rice: Insights into Volatile Flavor Compounds
Source: Foods. 2024 Oct 29;13(21):3453. doi: 10.3390/foods13213453 (PMC11545351; doi:10.3390/foods13213453)
Supplement: Supplementary file 1 [file foods-13-03453-s001.zip › foods-3261671-supplementary.pdf]

Table S1 Detailed rating rules and record form for sensory evaluation of cooked black rice (modified by GB/T 15682-2008 [25]).

**Primary attributes**	**Secondary attributes**	**Description: score**
Smell/20	-	A rich aroma of black rice: 18-20<br>A fairly aroma of black rice: 15-17<br>A slightly aroma of black rice: 12-14<br>No aroma but no bad smell: 7-12<br>Bad smell: 0-6
Appearance/20	Color/7	Unique pure color of black rice: 6-7<br>Normal color: 4-5<br>Abnormal yellowing or graying: 0-3
	Gloss of rice surface/8	Obvious gloss: 7-8<br>Slightly gloss: 5-6<br>Little gloss: 0-4
	Rice integrity/5	Tight and complete structure: 4-5<br>Mostly tight and complete structure: 3<br>Loose and incomplete structure: 0-2
Texture/30	Adhesiveness/10	Smooth, sticky, non-stick teeth: 8-10<br>Sticky, basically non-stick teeth: 6-7<br>Sticky, sticky to teeth; or no stickiness: 0-5
	Springiness/10	Chewy texture: 8-10<br>Slightly chewy texture: 6-7<br>Loose or hard texture: 0-5
	Hardness/10	Moderate hardness: 8-10<br>Slightly hard or slightly soft: 6-7<br>Very hard or very soft: 0-5
Taste/25	-	Slightly bitter taste of black rice: 22-25<br>Fairly bitter taste of black rice: 18-21<br>Obviously bitter taste of black rice, but No bad taste: 16-17<br>Bad taste: 0-15
Cold rice texture/5	-	Good viscoelasticity and moderate hardness: 4-5<br>Agglomeration, slightly poor viscoelasticity, slightly hardness: 2-3<br>Very poor viscoelasticity and hard: 0-1
Score for all sensory attributes/100		
